# Engineering the substrate binding site of the hyperthermostable archaeal endo-β-1,4-galactanase from *Ignisphaera aggregans*

**DOI:** 10.1186/s13068-021-02025-6

**Published:** 2021-09-16

**Authors:** Sebastian J. Muderspach, Folmer Fredslund, Verena Volf, Jens-Christian Navarro Poulsen, Thomas H. Blicher, Mads Hartvig Clausen, Kim Krighaar Rasmussen, Kristian B. R. M. Krogh, Kenneth Jensen, Leila Lo Leggio

**Affiliations:** 1 Department of Chemistry, University of Copenhagen, Universitetsparken 5, 2100 Copenhagen, Denmark; 2 Novozymes A/S, Biologiens vej 2, 2800 Kongens Lyngby, Denmark; 3 Center for Nanomedicine and Theranostics, Department of Chemistry, Technical University of Denmark, Kemitorvet 207, 2800 Kgs. Lyngby, Denmark; 4 Present Address: The Novo Nordisk Foundation Center for Biosustainability, Technical University of Denmark, 2800 Kgs. Lyngby, Denmark; 5 Present Address: Department of Genetics, Harvard Medical School, Boston, MA USA

**Keywords:** *Ignisphaera aggregans*, Archaea, Glycoside hydrolase, Biomass degradation, Extreme thermophile, Degradation profiles, Crystal structure, High-performance anion-exchange chromatography, Galactan, Rational design

## Abstract

**Background:**

Endo-β-1,4-galactanases are glycoside hydrolases (GH) from the GH53 family belonging to the largest clan of GHs, clan GH-A. GHs are ubiquitous and involved in a myriad of biological functions as well as being widely used industrially. Endo-β-1,4-galactanases, in particular hydrolyse galactan and arabinogalactan in pectin, a major component of the primary plant cell wall, with important functions in plant defence and application in the food and other industries. Here, we explore the family’s biological diversity by characterizing the first archaeal and hyperthermophilic GH53 galactanase, and utilize it as a scaffold for engineering enzymes with different product lengths.

**Results:**

A galactanase gene was identified in the genome of the anaerobic hyperthermophilic archaeon *Ignisphaera aggregans*, and the isolated catalytic domain expressed and characterized (IaGal). IaGal presents the typical (βα)_8_ barrel structure of clan GH-A enzymes, with catalytic carboxylates at the end of the 4th and 7th barrel strands. Its activity optimum of at least 95 °C and melting point over 100 °C indicate extreme thermostability, a very advantageous property for industrial applications. If enzyme depletion is reduced, so is the need for re-addition, and thus costs. The main stabilizing features of IaGal compared to other structurally characterized members are π–π and cation–π interactions. The length of the substrate binding site—and thus produced oligosaccharide products—is intermediate compared to previously characterized galactanases. Variants inspired by the structural diversity in the GH53 family were rationally designed to shorten or extend the substrate binding groove, in order to modulate product length. Subsite-deleted variants produced shorter products than IaGal, as do the fungal galactanases inspiring the design. IaGal variants engineered with a longer binding site produced a less expected degradation pattern, though still different from that of wild-type IaGal. All variants remained extremely stable.

**Conclusions:**

We have characterized in detail the most thermophilic endo-β-1,4-galactanase known to date and successfully engineered it to modify the degradation profile, while maintaining much of its desirable thermostability. This is an important achievement as oligosaccharide products length is an important property for industrial and natural GHs alike.

**Supplementary Information:**

The online version contains supplementary material available at 10.1186/s13068-021-02025-6.

## Background

Endo-β-1,4-galactanases degrade galactan and arabinogalactan, which are found in the rhamnogalacturonan I part of pectin abundant in the cell wall of non-woody plants. The backbone of rhamnogalacturonan I consists of alternating galacturonic acid and rhamnose. The rhamnose is occasionally decorated with long chains of β-1,4-linked galactan, arabinan and arabinogalactan [1, 2]. Pectin is a versatile polysaccharide and in the plant it is important for both the growth of the cells and as a defence mechanism against plant pathogens [3, 4]. Pectin has long been used in the food industry as a thickening agent [5], but manipulation of the pectin network has recently been of interest in other fields such as controlled fruit ripening [6] and in medical industry both as cancer treatment [7] and in drug delivery systems [8].

Endo-β-1,4-galactanases are glycoside hydrolases (EC 3.2.1.89) that degrade the galactan and arabinogalactan via a double displacement retaining mechanism using two carboxylic acids, one acting as a nucleophile and one functioning as an acid/base. Endo-β-1,4-galactanases are classified in the CAZy database as GH53 [9] which is part of the largest clan to date, the GH-A clan [10, 11]. Galactanases can be used in conjunction with other enzymes for biomass degradation to create small building blocks or biofuel via fermentation [12], or as additives to improve digestibility of animal feed [13], and in the production of prebiotic oligosaccharides for functional foods [14]. In fact, endo-β-1,4-galactanases are a part of the machinery used by probiotic organisms to process lactose-derived galactooligosaccharides [15]. To date, four fungal endo-β-1,4-galactanases from the following sources have been structurally and biochemically characterized: *Thermothelomyces thermophila* (also known as *Myceliophthora thermophila*) (MtGal, GenBank ID AAE73520.1, PDB IDs: 1HJS, 1HJU) [16], *Humicola insolens* (HiGal, GenBank ID AAN99815.1, PDB ID: 1HJQ) [16], *Aspergillus aculeatus* KSM 510 (AaGal, GenBank ID AAA32692.1, PDB IDs: 1FHL, 1FOB) [17] and the highly related *Aspergillus nidulans* FGSC A4 (EnGal, GenBank ID ABF50874.1, PDB ID: 4BF7) [18]. Two bacterial endo-β-1,4-galactanase have also been structurally characterized, one from *Bacillus licheniformis* (BlGal, GenBank ID AAO31370.1, PDB IDs: 1R8L, 1UR0, 1UR4, 2CCR, 2GFT, 2J74) [19, 20], and one from *Bacteroides thetaiotaomicron* (BtGal, GenBank ID AAO79773.1, PDB IDs: 6GP5, 6GPA) [21]. The fungal galactanases mentioned above as well as BtGal can degrade the galactan substrate down to galactobiose [15, 20, 21]. In addition, some fungal galactanases can carry out trans-glycosylation, unlike BlGal which neither degrades galactotriose nor performs trans-glycosylation [20, 22]. This has been ascribed to an extended loop providing − 3 and − 4 binding subsites giving BlGal the ability to bind the triose non-productively and is further supported by crystal structures of BlGal with small galactooligosaccharides bound from the − 2 to − 4 subsites (PDB IDs: 1URO, 1UR4, 2CCR, 2GFT, 2J74) [19, 20].

A structure of AaGal in complex with galactobiose has been determined (PDB ID 6Q3R), elucidating binding at the − 1 and − 2 subsites [23]. This binding mode agrees with the observed degradation patterns of fungal galactanases seen in Ryttersgaard et al*.* [20] where galactotetraose and galactotriose were degraded completely to galactose and galactobiose.

The temperature activity optimum on β-1,4-galactans has been reported for several galactanases. AaGal, EnGal and BlGal (on potato galactan) are mesophilic galactanases with temperature activity optima of 50 °C (on pectic galactan and AZCL-galactan), 49 °C (on potato galactan) and 40 °C (on potato galactan), respectively [12, 16, 24–26], while MtGal and HiGal are thermophilic with temperature optima of 65 °C, both measured using AZCL-lupin galactan as substrate [16]. To expand our knowledge beyond mesophilic and thermophilic endo-β-1,4-galactanases, we looked into hyperthermophilic archaea based on the hypothesis that a high-temperature environment drives an evolutionary adaptation of the proteome towards temperature stability. In 2006, the hyperthermophilic archaea *Ignisphaera aggregans* with an optimal growth temperature of 92–95 °C at slightly acidic conditions (pH 6.4) was discovered [27]. *I. aggregans* belongs to the crenarchaeota phylum containing a multitude of other well-known hyperthermophilic genus, such as *Sulfolobus* and *Thermofilum*. What makes *I. aggregans* particularly interesting is that it is one of only two archaeal genus’ encoding GH53 endo-β-1,4-galactanases, *Desulfurococcus* being the second one. In this article, we thus present the structure and characterization of the hyperthermophilic endo-β-1,4-galactanase catalytic domain from *I. aggregans* (IaGal). This catalytic domain, obtained from the C-terminal truncation of the full-length gene also encoding an Ig-like and a putative binding domain exhibits a temperature activity optimum of 95 °C making it an asset for future industrial biomass degradation. To increase the applicability of this hyperthermophilic enzyme, four variants of IaGal were produced and characterized. The variants were designed to resemble the fungal galactanases or BlGal in terms of binding sites and product profiles, while maintaining the exceptional stability of the parent enzyme.

## Results

### IaGal is a highly thermostable and thermophilic galactanase

**Fig. 1:**
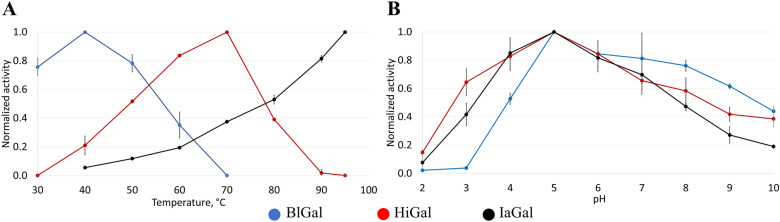
Activity optima for native endo-β-1,4-galactanases. **A** Normalized temperature activity optimum of BlGal, HiGal and IaGal at pH 5.0. **B** Normalized pH activity profile of BlGal, HiGal and IaGal at 40 °C. All experiments were done in triplicates and the error bars represent the standard deviations of the normalized activity

Initial analysis of IaGal showed a temperature activity optimum of at least 95 °C (in 50 mM NaOAc pH 5.0) and a pH optimum of 5.0 at a temperature of 40 °C with lupin galactan as substrate. For comparison, BlGal and HiGal showed a similar pH optimum, but a temperature activity optimum of approximately 40 °C and 70 °C, respectively, (see Fig. 1) in agreement with previous studies [12, 16].

Differential scanning calorimetry (DSC) indicated a thermal denaturation midpoint (*T*_m_) of 105 °C for IaGal making it to our knowledge the most thermostable galactanase ever reported. For reference BlGal showed a *T*_m_ of 53 °C, HiGal showed a *T*_m_ of 75 °C (see Additional file 1: Figure S1) and the *T*_m_ of AaGal has previously been measured at 61 °C [28].

### Comparison of IaGal galactan degradation profile to well-characterized galactanases

Due to the technical difficulties of measuring at temperatures close to its optimum, further assays for IaGal and its variants were carried out at 70 °C. According to the temperature optimum studies, the enzyme should retain 40% of the maximum enzymatic capabilities at this temperature. Lupin galactan was the substrate of choice due to its high content of long, unbranched galactan chains, but IaGal also proved active on potato galactan and AZCL-galactan (data not shown).

Michaelis–Menten analyses for IaGal (WT and variants) and the references MtGal and BlGal are shown in Table 1. For the IaGal WT the *K*_m_ is 0.47 mg ml^−1^ and *k*_cat_ is 34.3 s^−1^. Thus, the catalytic efficiency of the enzyme measured at pH 5.0 and 70 °C is 72.8 ml mg^−1^ s^−1^. The catalytic efficiencies for the reference galactanases MtGal and BlGal are 347.8 ml mg^−1^ s^−1^ and 263 ml mg^−1^ s^−1^, respectively (at 30 °C), but one should be careful when directly comparing catalytic efficiencies of enzymes far from the temperature activity optimum.

**Table 1:** Michaelis–Menten kinetic parameters and thermal denaturation points

	*K*_m_ (mg ml^−1^)	*k*_cat_ (s^−1^)	*k*_cat_/*K*_m_$(\mathrm{ml }\,{\mathrm{mg}}^{-1}\, {\mathrm{ s}}^{-1})$	*T*_m_ (°C)
IaGal WT	0.47 ± 0.08	34 ± 2	73 ± 13	105
Variant 1	0.38 ± 0.09	14 ± 4	36 ± 13	102
Variant 2	0.48 ± 0.08	16 ± 3	34 ± 9	101
Variant 3	2.3 ± 0.6	28 ± 15	12 ± 7	103
Variant 4	2.7 ± 0.3	20 ± 6	7 ± 2	103
BlGal	0.94 ± 0.15	2.5·10^2^ ± 0.1·10^2^	2.6·10^2^ ± 0.4·10^2^	53
MtGal	1.0 ± 0.4	3.6·10^2^ ± 0.3·10^2^	3.5·10^2^ ± 1.3·10^2^	N/A

The parameters are on lupin galactan in 50 mM NaOAc pH 5.0 and 0.01% Triton X-100, at 70 °C for IaGal WT and variants and 30 °C for BlGal and MtGal. The 95% confidence interval is calculated for the parameters. The measured thermal denaturation midpoint (*T*_m_) is presented for comparison

**Fig. 2:**
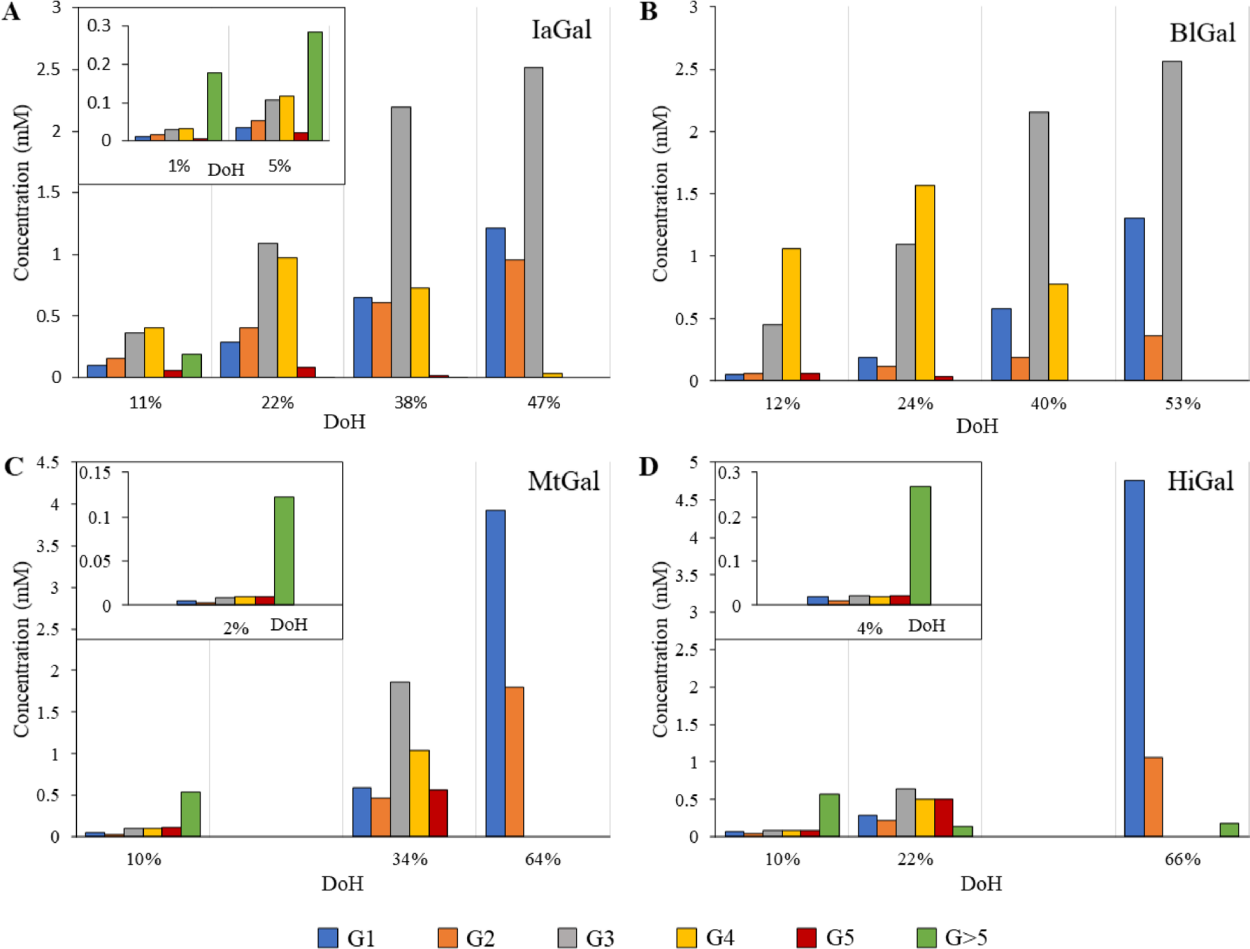
Degradation profiles for native endo-β-1,4-galactanases. The degradation products of IaGal (**A**), BlGal (**B**), MtGal (**C**) and HiGal (**D**) hydrolysing lupin galactan are shown. The concentrations of G1 (galactose), G2 (galactobiose), G3 (galactotriose), G4 (galactotetraose), G5 (galactopentaose) and G > 5 (larger than galactopentaose) are reported in mM for each given DoH (shown on the x-axis). The %DoH is defined as the amount of reducing ends divided by the theoretical maximum amount of reducing ends, i.e. a full degradation to galactose

The detailed degradation profiles for IaGal and the reference enzymes (shown in Fig. 2) were measured using high-performance anion exchange chromatography coupled with pulsed amperometric detection (HPAEC-PAD) and a 4-hydroxybenzhydrazide (PAHBAH) assay. To indicate where the degradation process of the given sample was stopped, a degree of hydrolysis (DoH) was calculated for each sample based on the results from the PAHBAH assay. The DoH is defined as the amount of reducing ends divided by the theoretical maximum amount of reducing ends, i.e. a full degradation to galactose. The amount of reducing ends measured by the PAHBAH assay compared to the amount of galactose (G1), galactobiose (G2), galactotriose (G3), galactotetraose (G4) and galactopentaose (G5) quantified using HPAEC-PAD gives a rough estimate of the amount of galactooligosaccharides larger than G5 (G > 5) present in the sample.

The initial degradation of lupin galactan by IaGal WT produces mainly G > 5, but G3 and G4 are also produced in significant amounts. At DoH 11% G > 5 is no longer the main constituent in terms of concentration and at DoH of 22% no G > 5 is observed. A higher DoH lead to an accumulation of G1, G2 and G3 in a molar ratio of 3:2:6 (Fig. 2A).

The bacterial galactanase BlGal has an extended substrate binding site compared to IaGal, while the fungal galactanases MtGal and HiGal have shorter binding sites. They were chosen as references as structures of them have been published. As previously observed, fungal galactanases produced all the quantifiable small oligosaccharides (from G1 to G5) at low DoH [20, 24, 29], but MtGal and HiGal initially produced G > 5 too. These larger components were both detected in the chromatograms (not shown) and clearly seen in the comparison of the reducing end assay and the HPAEC-PAD analyte quantification (see Fig. 2). Both HiGal and MtGal appear to produce less G2 than other analytes at 10% DoH and the larger G > 5 products appear to be degraded primarily to G3 and G4. At the end of the hydrolysis G3, G4 and G5 are degraded to G1 and G2 in molar ratios of 2:1 for MtGal and 4:1 for HiGal. It is unknown whether the residual G > 5 observed for HiGal is caused by an artifact or if the enzyme is unable to degrade the lupin galactan fully. At high DoH both fungal enzymes produced an easily quantifiable peak eluting close to the G2 peak, which was also observed in the degradation profile for AaGal in Ryttersgaard et al*.* [20]. We confirmed the peak to correspond to a β-1,3-galactobiose trans-glycosylation product using the retention time of standards.

The degradation pattern for BlGal is also consistent with previous results [20], proving the enzyme able to degrade G4 but not G3. BlGal initially produces large amounts G4 and a significant amount of G3, but only miniscule amounts of G5 is detected and no compounds larger than G5 is seen either inspecting the chromatograms or the comparison between the reducing end assay and the HPAEC-PAD results. The substrate is degraded to G1, G2 and G3 in a 3:1:6 ratio, which again is consistent with previous results.

### 3-Dimensional structure of IaGal

**Fig. 3:**
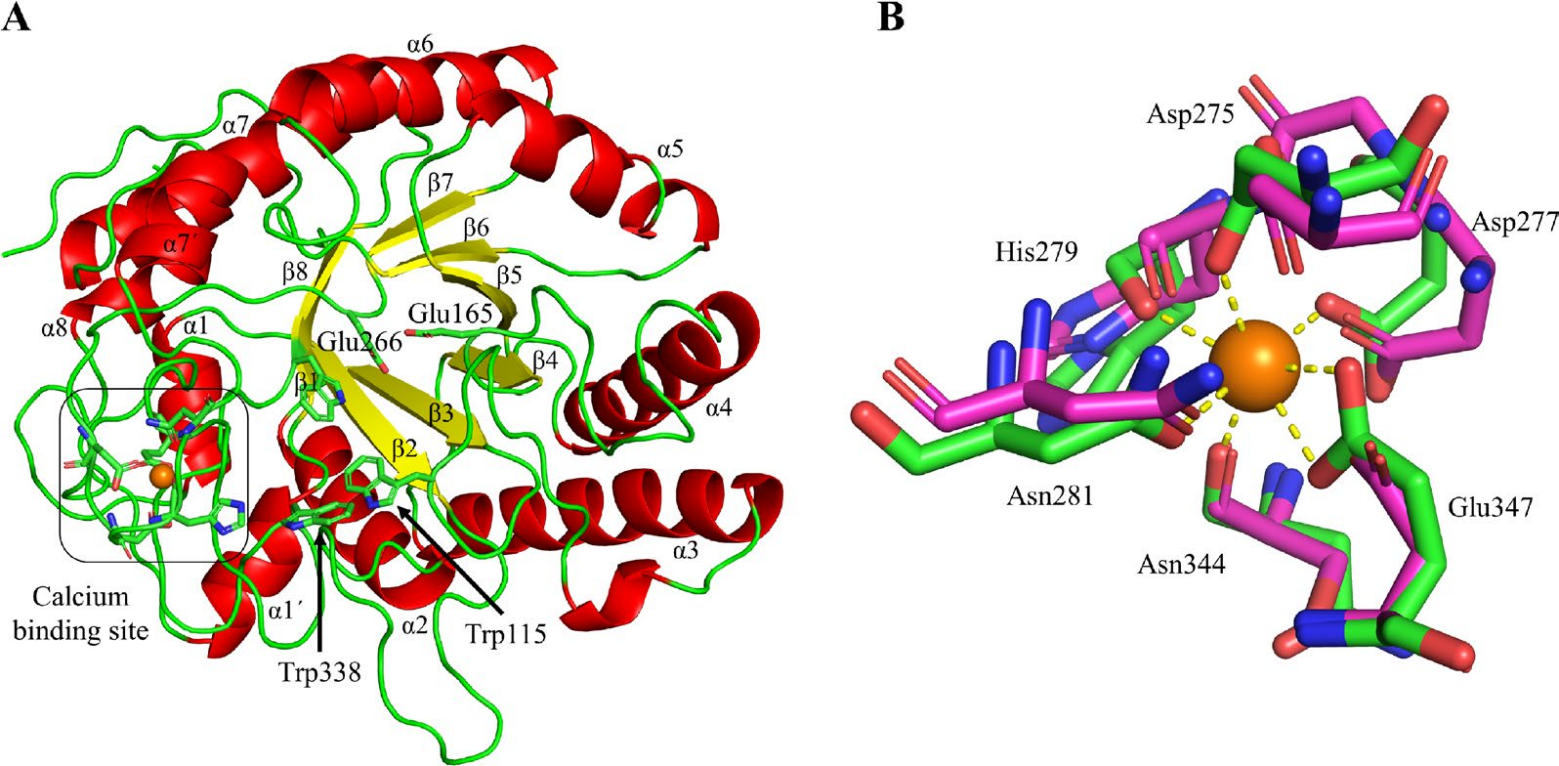
Overview of the IaGal structure. **A** The overall structure of the IaGal catalytic domain. Secondary structural elements are coloured (α-helices red, and β-sheets yellow). The catalytic acid/base (Glu165) and catalytic nucleophile (Glu266) are shown in sticks. Trp115 and Trp338 correspond to the − 2 and − 3 subsites, respectively. The calcium binding site is also highlighted. **B** The calcium binding site of IaGal (PDB ID: 7OSK, green) compared to that from the bacterial enzyme BlGal (PDB ID: 1UR0, magenta)

The structure of IaGal was determined by X-ray crystallography and the statistics are given in Additional file 1: Table S1. As other enzymes in the GH53 family and clan GH-A, the overall structure of IaGal is a (β/α)_8_ barrel (also known as TIM barrel) which consists of 8 parallel β-sheets (here referred as β1 to β8) and 8 α-helices (here referred as α1 to α8) that alternate along the peptide chain. Loops connecting βn and αn will be referred to the nth β/α-loop. β4 and β7 bear the catalytic acid–base (Glu165) and nucleophile residues (Glu266), respectively (see Fig. 3). All galactanases structurally characterized to date contain a stabilizing interaction between the 7th and 8th β/α-loop in the (β/α)_8_ barrel except for BtGal, which has a significantly shorter 7th β/α-loop. The stabilizing interaction has been shown to be a disulphide bridge in all fungal galactanases, and a calcium binding site in BlGal (purple) and now archaeal (green) enzymes (Fig. 3). A slight difference is observed in the calcium binding sites between BlGal and IaGal as Glu347 in the archaeal enzyme coordinates both with Oε1 and Oε2, while the corresponding Asp370 in BlGal is angled differently and only coordinates with one oxygen. This results in a coordination number for the calcium ion of 7 in IaGal compared to 6 in BlGal. The difference is likely occurring due to the disparity in pH of the crystallization conditions as BlGal was crystallized in pH 5.0 and IaGal was crystallized in pH 7.5.

### Structural features related to thermal adaptation

Since almost all structurally characterized galactanases have stabilizing features between loops 7 and 8 (a calcium binding site or a disulphide bridge), other structural features must be responsible for the much larger thermal stability and thermophilicity of IaGal. The structural features responsible for thermal adaptation of proteins can vary, as reviewed for example in [30, 31], thus various features were analysed for all structurally characterized GH53 enzymes and Pearson correlation coefficients of temperature optimum with each of the given features calculated (Additional file 1: Table S2). For most of these, experimental activity temperature optima are known, with the exception of BtGal (for which a temperature optimum was arbitrarily set at 37 °C due to its human gut niche), thus correlation coefficients were calculated excluding BtGal from the analysis. The features analysed include various types of electrostatic interactions (salt-bridges, charge stabilization/destabilization of helix dipoles), specific amino acid residues content, and features indicative of the compactness of the protein (surface/volume ratio). Cation–π and π–π interactions show highest correlation with thermophilicity in the GH53 family, with CCs of 0.74/0.70 and 0.94/0.94, respectively, with/without inclusion of BtGal in the analysis. Helix stabilization, salt bridges and Pro/Gly ratio may also contribute to stabilization of IaGal, but are not as strongly correlated with thermophilicity.

### IaGal has a substrate binding groove with intermediate features compared to previously known galactanases

**Fig. 4:**
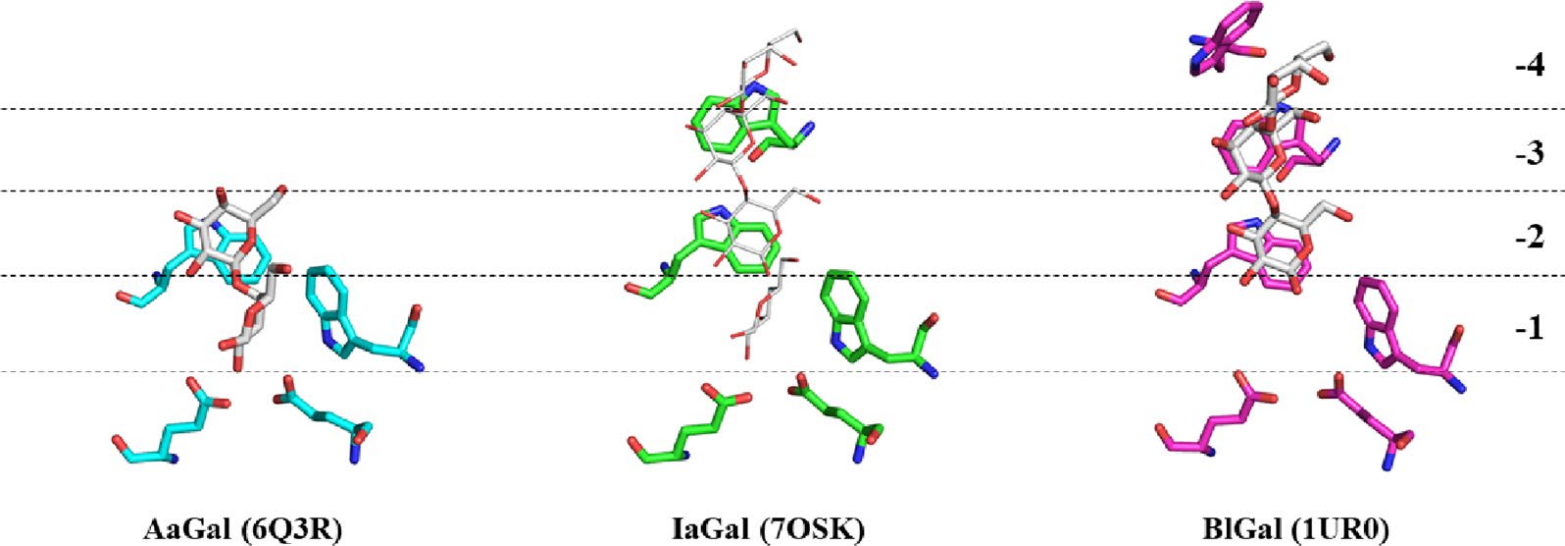
Substrate binding sites of AaGal, IaGal and BlGal. Substrate subsites at the non-reducing end increase incrementally from fungal enzymes (AaGal PDB ID 6Q3R) to archaeal enzymes (IaGal PDB ID 7OSK) to the bacterial BlGal enzyme (PDB ID 1UR0). The galactobiose and galactotriose ligands are shown as white sticks in the AaGal and BlGal structures, respectively. The same ligands are shown as white lines after superposition onto the IaGal structure

It was not possible to obtain experimental complexes of oligosaccharides with IaGal since well-diffracting crystals were only rarely obtained. Fortunately, binding of galactooligosaccharides to BlGal has previously been characterized structurally and correlates well with the degradation pattern differences seen between fungal galactanases and BlGal as reiterated in a previous section. From the structural point of view, IaGal can be considered an intermediate with respect to substrate binding subsites (Fig. 4 and Additional file 1: Figure S2). The IaGal contains − 1, − 2 and − 3 binding subsites in addition to the + subsites, while the fungal galactanases are devoid of the − 3 subsite and BlGal contains the additional − 4 subsite.

### Design and production of stable IaGal variants with extended and shortened substrate binding sites

Given the extreme thermostability of IaGal, it was of interest to create variants through rational engineering, which could mimic degradation profiles of other less thermostable GH53 galactanases. These alterations to the degradation profiles make the enzyme more amenable for downstream applications such as ethanol production while still retaining the extreme thermostability allowing for a broader range of potential industrial processes.

**Fig. 5:**
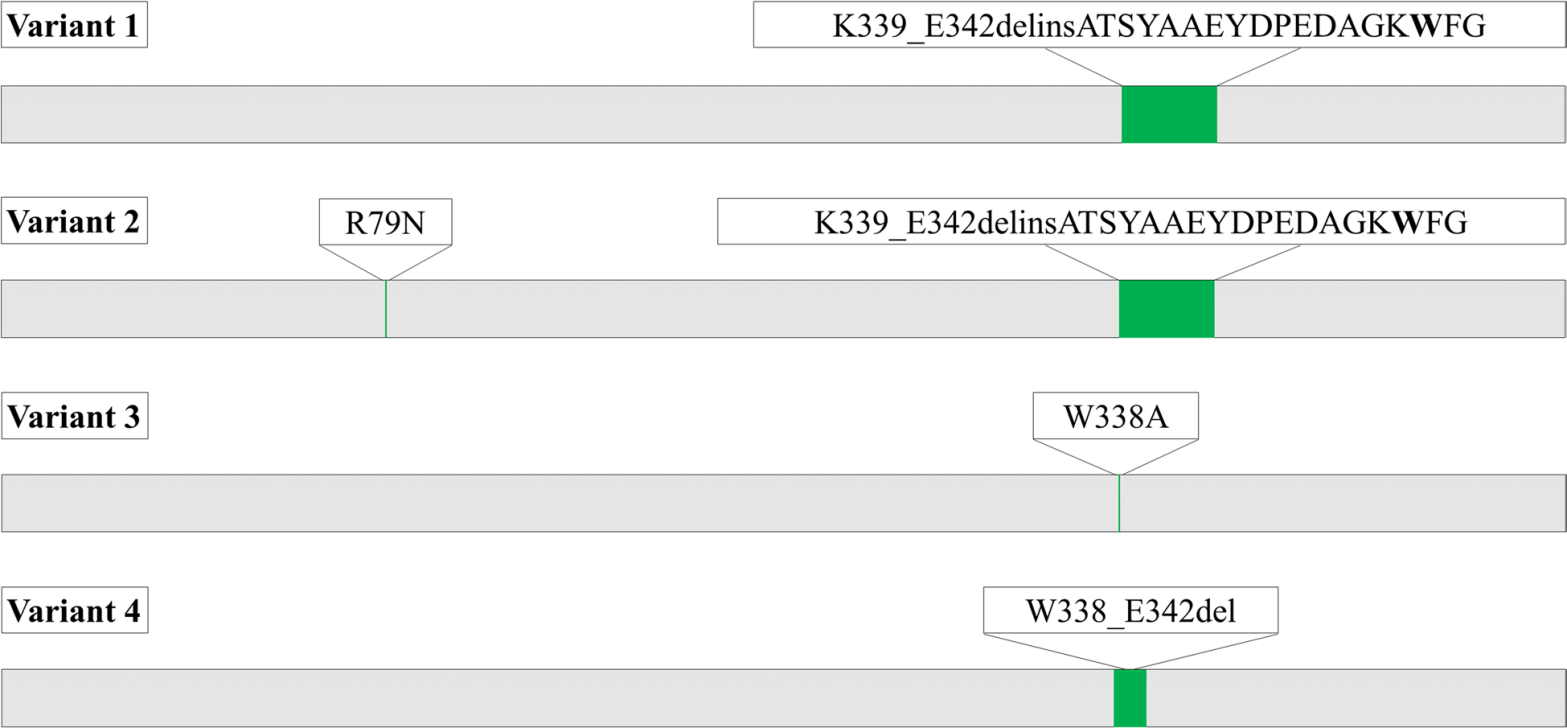
IaGal variant overview. In subsite-extended variant 1 and 2 the residues 339–342 were replaced by the sequence ATSYAAEYDPEDAGKWFG with variant 2 having an additional point mutation (R79N). The tryptophan intended as the aromatic platform for binding subsite − 4 is highlighted in bold. Subsite-deleted variant 3 is a point mutation (W388A) while in variant 4 the residue sequence 338–342 has been deleted. The variant nomenclature in the figure is adopted from [32]

Two variants, 1 and 2, were constructed to resemble a bacterial galactanase (BlGal) by adding the − 4 binding subsite and are referred to in the text to follow as subsite-extended variants. Another two variants, 3 and 4, were constructed to resemble the fungal galactanases by deleting the − 3 binding subsite; these are referred to as subsite-deleted variants. In the subsite-extended variants, the loop sequence from A348 to G365 (based on BlGal) is added between W338 and G343 deleting a small part of the IaGal loop (see Fig. 5). For variant 2, the additional point mutation R79N is constructed as this arginine could be sterically hindering the correct position of the inserted loop. In the subsite-deleted variants the − 3 subsite is impaired by a point mutation (W338A) of the aromatic platform (variant 3) or the whole loop W338 to E342 is deleted (variant 4). All variants could be produced and remained highly thermostable with *T*_m_ between 101 and 103 °C (Table 1), which is only a slight decrease in thermostability from the native IaGal catalytic domain.

**Fig. 6:**
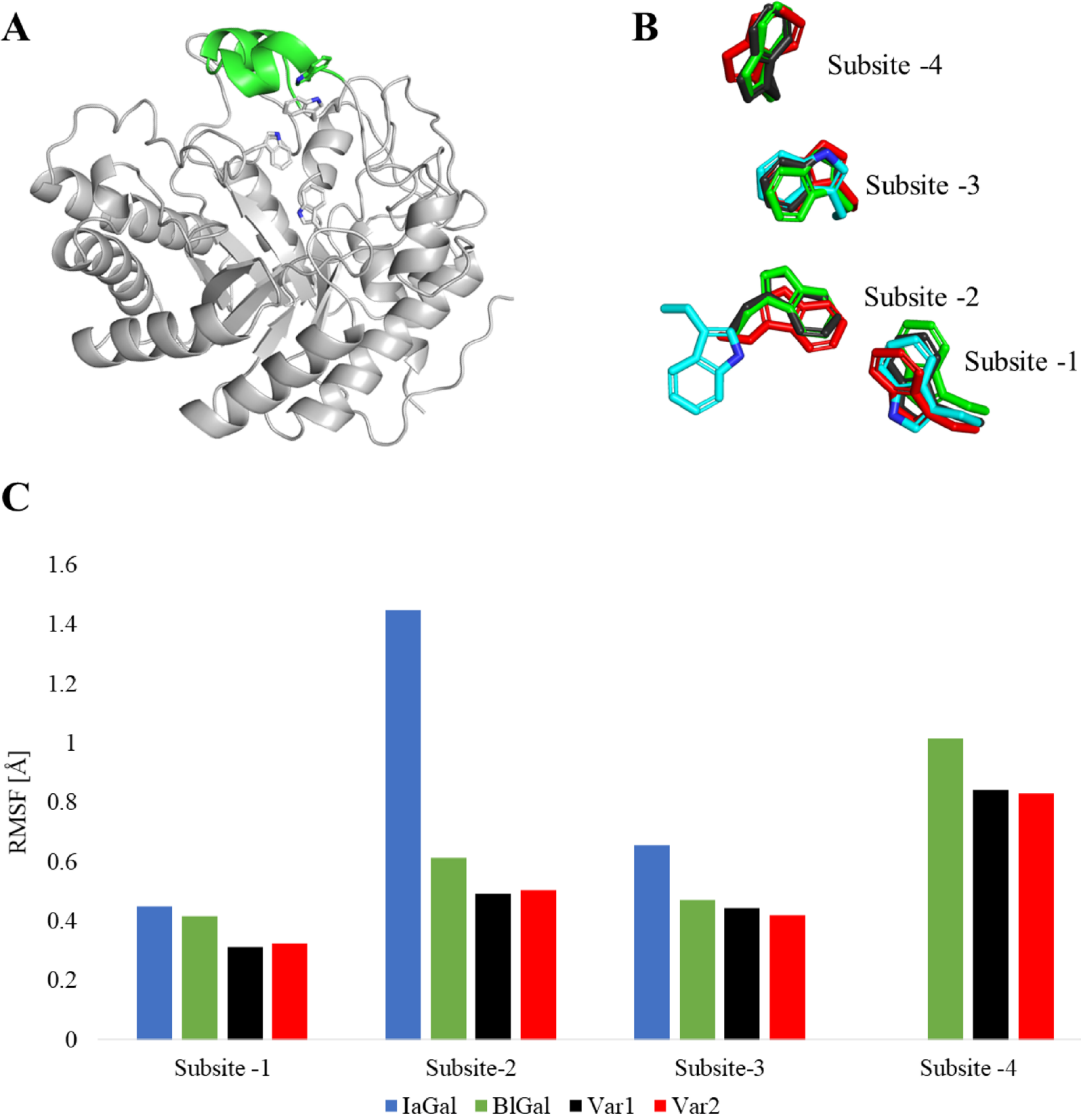
Molecular dynamic simulations of subsite-extended variants. **A** Overview of variant 1 after 90 ns of simulation. The inserted sequence is shown in green and the platforms for the non-reducing end binding subsites are shown as sticks. **B** Comparison of the non-reducing end binding subsites for variant 1 (black), variant 2 (red) and IaGal (blue) after 90 ns simulation and BlGal (PDB ID 1R8L, green). **C** Comparison of the RMSF for the non-reducing end binding subsites using the same colour scheme

**Fig. 7:**
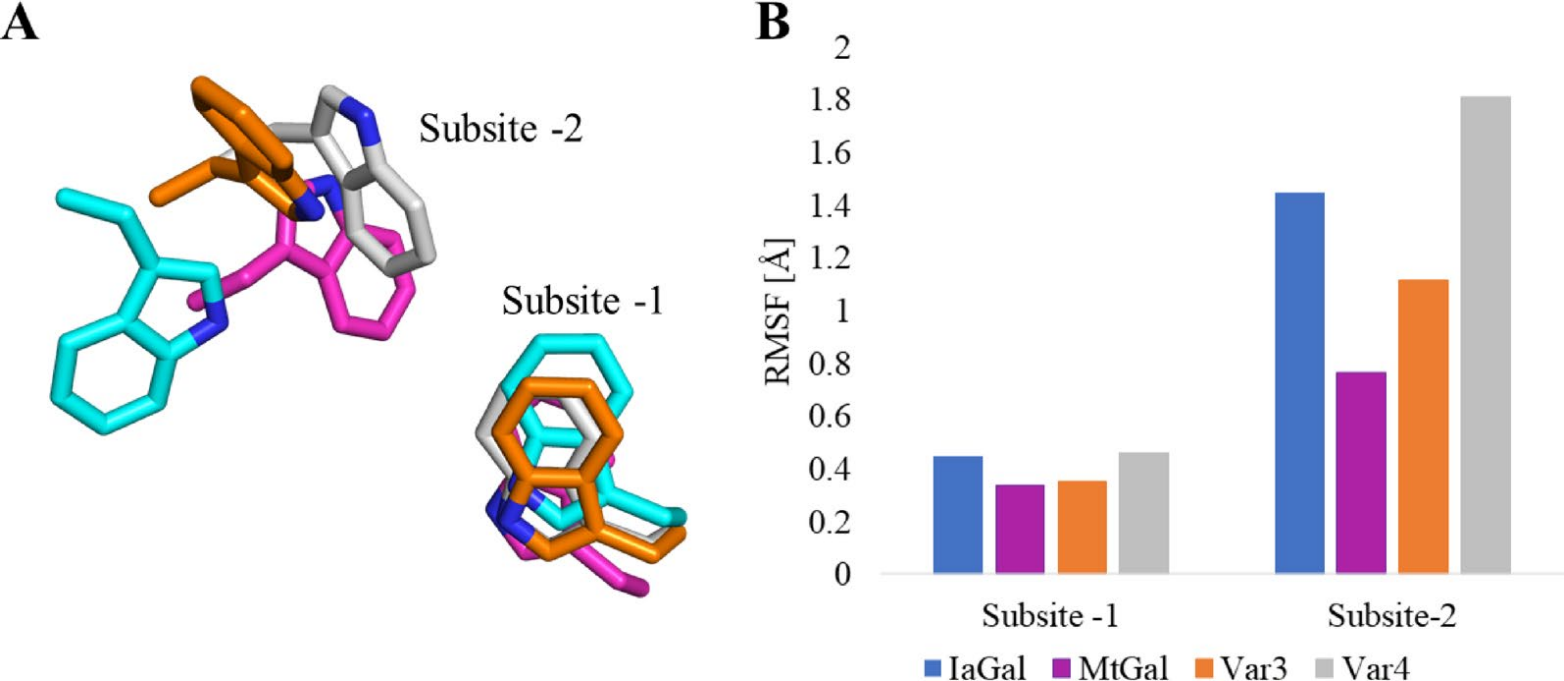
Molecular dynamic simulations of subsite-deleted variants. IaGal is shown in blue, MtGal is magenta, variant 3 is orange and variant 4 is grey. A Comparison of the − 1 and − 2 binding subsites for variant 3, variant 4, IaGal and MtGal after 90 ns of simulation. B Comparison of the RMSF value for the binding subsites

As crystallization of the variants did not succeed, homology models were created. Variants 1 and 2 were created using both BlGal and IaGal as templates. Variant 3 was based solely on IaGal while variant 4 used both IaGal and MtGal as templates. The variant homology models as well as the crystal structures of IaGal, BlGal and MtGal were subjected to molecular dynamics simulations. To indicate the fluctuation of the residues, the root mean square fluctuation (RMSF) was calculated for all residues over the full length of the simulations. RMSF is the root mean square deviation of the residue’s coordinates from a reference structure (the first structure of the simulation in this case) averaged over time. As seen in Fig. 6, the added loop in variants 1 and 2 is correctly formed and assumes the same conformation as seen in BlGal (PDB ID 1R8L), remaining stable after 90 ns of simulation. The binding subsites are centred on the side chain indole of a Trp residue, and the stability of the subsite is assessed by monitoring the corresponding Trp. The Trp of the added binding subsite does have a higher RMSF value compared to the other binding subsites, but the value is lower in variants 1 and 2 compared to the simulation of the parent BlGal (PDB ID 1R8L) itself. Interestingly the Trp corresponding to the − 2 binding subsite of IaGal appears to alternate between two conformations which is the cause of the high RMSF value. The subsite-deleted variants also have a less stable − 2 binding subsite where the Trp in both cases flips 90° after approximately 50 ns (see Fig. 7). This flip is not observed for the fungal galactanase MtGal nor the subsite-extended variants nor is the flip similar to the IaGal flip. RMSF plots for all residues are seen in Additional file 1: Figure S3.

### Characterization of galactan degradation by subsite-extended and subsite-deleted IaGal variants

The kinetic parameters *K*_m_, *k*_*cat*_ and the catalytic efficiency for the IaGal WT and the variants are listed in Table 1, showing a decrease in catalytic efficiency for all variants. The decrease is twofold for subsite-extended variants 1 and 2 and 6- to tenfold for subsite-deleted variants 3 and 4. This large decrease in catalytic efficiency for the subsite-deleted variants is mainly caused by a large increase in *K*_m_. This is an expected increase as the binding site is weakened by removing the − 3 binding subsite, but is further emphasized by the results of the simulations indicating a less structurally stable − 2 binding subsite.

**Fig. 8:**
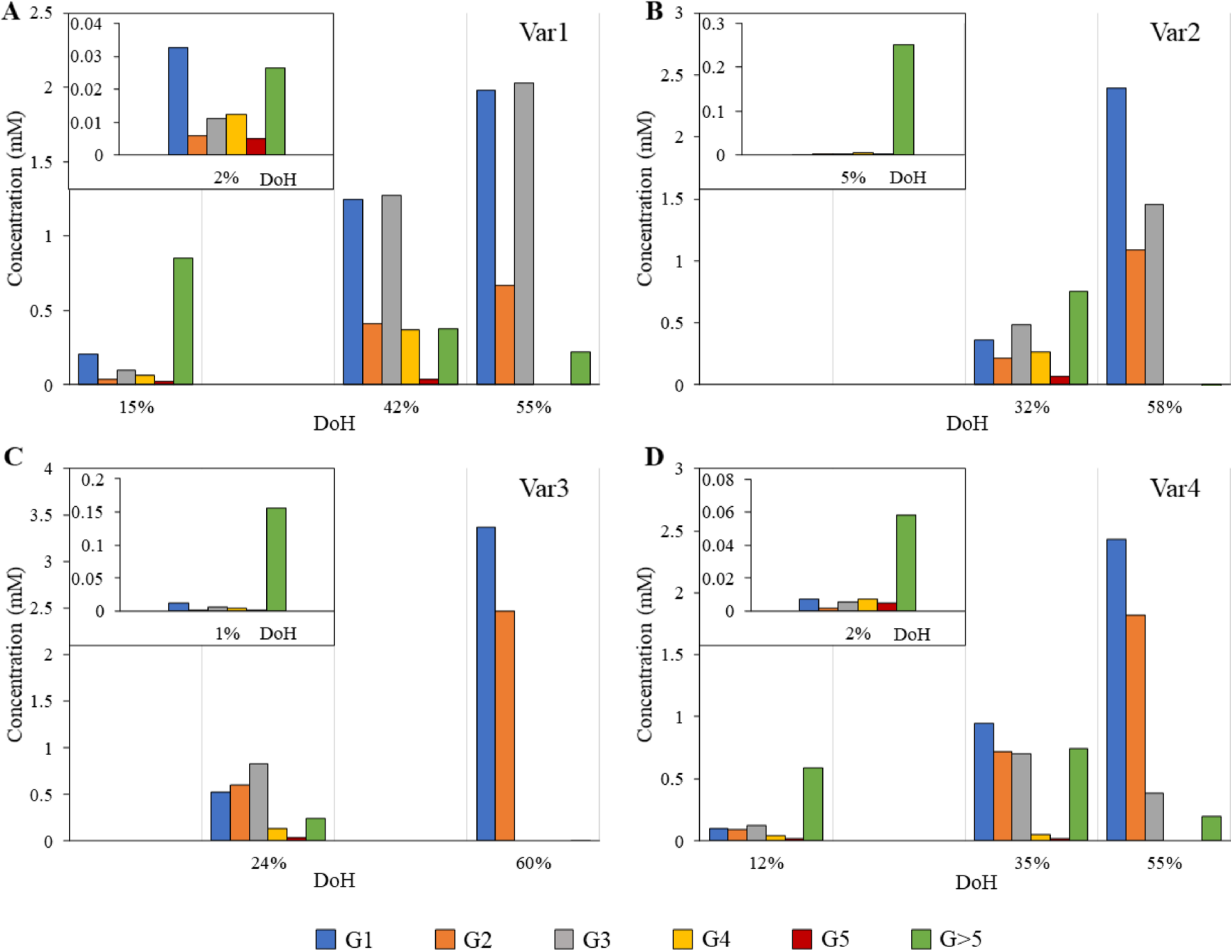
Degradation profiles of IaGal variants. The degradation products of variant 1 (**A**), variant 2 (**B**), variant 3 (**C**) and variant 4 (**D**) hydrolysing lupin galactan are shown. The concentrations of G1 (galactose), G2 (galactobiose), G3 (galactotriose), G4 (galactotetraose), G5 (galactopentaose) and G > 5 (larger than galactopentaose) are reported in mM for each given DoH. The %DoH is defined as the amount of reducing ends divided by the theoretical maximum amount of reducing ends, i.e. a full degradation to galactose

The degradation patterns of the two subsite-deleted variants 3 and 4 are similar to those of MtGal and HiGal, as both initially produced galactooligosaccharides in varying sizes but the main product produced was G > 5 (see Fig. 8). Both variants likewise showed ability to degrade the substrate to G1 and G2 in molar ratios of 3:2. Interestingly, the subsite-deleted variants obtained the ability to transglycosylate to β-1,3-galactobiose at high DoH as was seen for MtGal and HiGal and previously observed for AaGal [20, 22]. Variant 1 initially produced large amounts of G1 but G3, G4, G5 and G > 5 were also present while the degradation pattern of variant 2 primarily showed production of G > 5 at low DoH. Both variant 1 and 2 degrade G4 to G1, G2 and G3, but in ratios that neither resemble BlGal nor IaGal. Variant 1 degrades to a G1:G2:G3 ratio of 2:1:2 while variant 2 degrades to a ratio of 5:2:3. Both therefore show tendencies to produce a lot more G1 compared to both IaGal and BlGal.

## Discussion

### Structural features contributing to IaGal thermal adaptation

IaGal has by far the highest temperature activity optimum (≥ 95 °C) of characterized galactanases and is remarkably thermostable with a *T*_m_ > 100 °C.

Several structural features have been reported to increase thermostability and thermophilicity of proteins in general, including increased Pro/Gly ratio, salt bridges, hydrogen bonds, π–π and cation–π interactions [31, 33–37]. Compactness and efficient packing, as indicated by parameters such as surface/volume ratio, have also been found relevant [38]. For GH53 galactanases, previous structural analysis [16] highlighted the importance of dipole stabilization of α-helices in the (βα)_8_ barrel and cation–π interactions based on the structures available at the time, with hydrogen bonds and ion pairs as secondary factors. In [28], an AaGal variant with a G306A mutation in the 8th β/α loop increased the *T*_m_ by 1.1 °C. In [39], a G305A variant in the same loop in TsGal showed a 8.6-fold increase in half-life at 55 °C. Thus, the 8th β/α-loop seems to be a hotspot for stabilizing mutations.

**Fig. 9:**
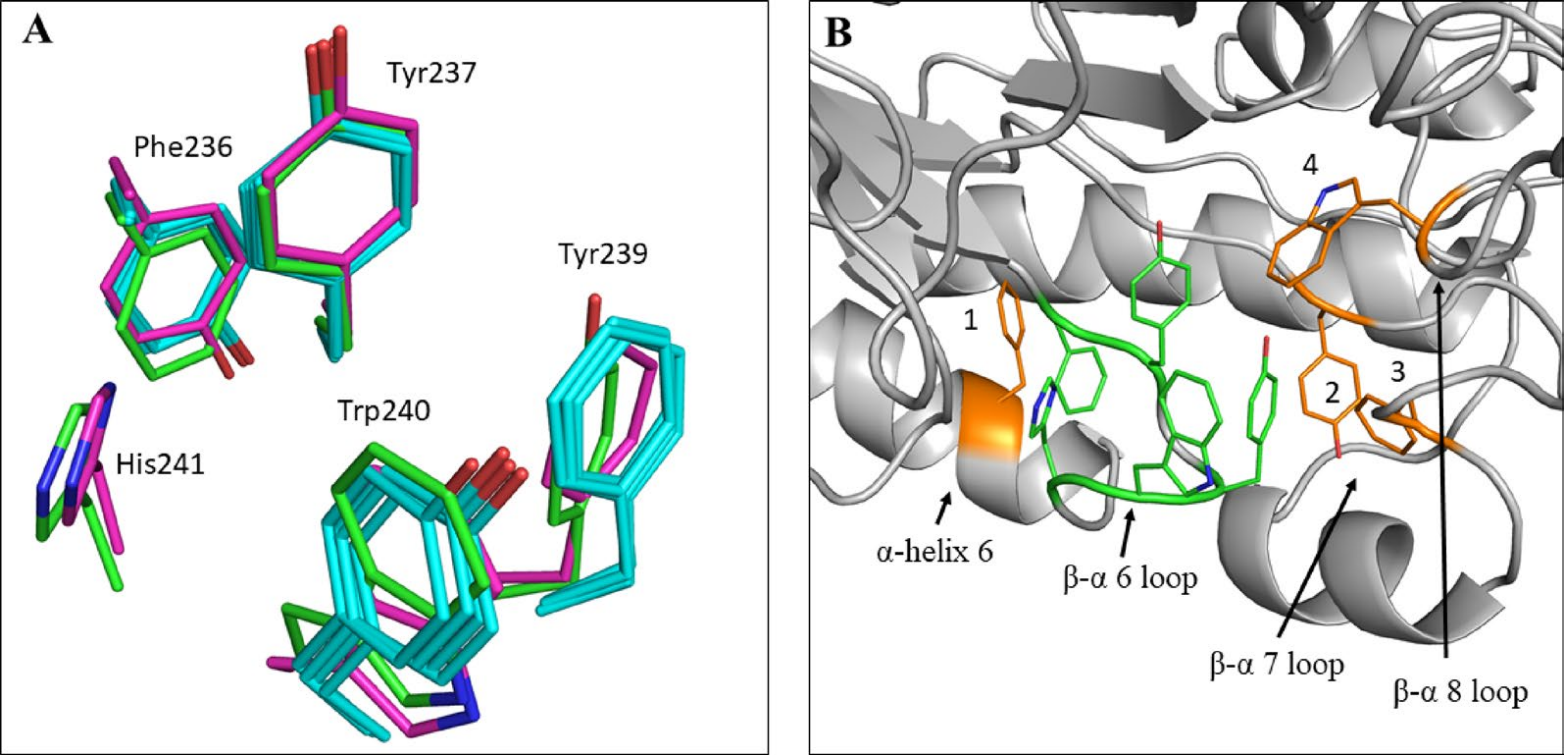
Highly conserved area containing π–π interactions in GH53 galactanases. **A** Superposition of the conserved aromatic residues localized in the 6th β/α-loop. The residues from IaGal are labelled and shown in green, BlGal is shown in magenta and all the fungal galactanases are shown in cyan. Unique for BlGal and IaGal is the extension of the aromatic cluster with an additional histidine residue. **B** An overview of the π–π cluster in IaGal. The residues located in the 6th β/α-loop are shown as greens sticks in the same orientation as in **A**. Adjacent aromatic residues which interact with these conserved residues are shown as orange sticks (1: Phe247, 2: Tyr269, 3: Phe283, 4: Trp346)

In light of the extreme thermostability of IaGal, we have here analysed again the available galactanase structures in GH53 (Additional file 1: Table S2) for stabilizing features. We find that, while salt-bridges and helix stabilization may contribute to IaGal thermostability, H-bonds and compactness do not, and cation–π and π–π interactions are the only features correlating strongly with thermophilicity in GH53 in general. As seen in Fig. 9, a relatively large portion of π–π interactions is centred around the 6th β/α-loop. This cluster of π–π interactions is a highly conserved region between all structurally determined galactanases except BtGal, which only contains a few aromatic residues in this area and even fewer π–π interactions due to a difference in orientation of the aromatic residues. The conserved cluster of aromatic residues appear not only to be stabilizing the 6th β/α-loop (uniquely in IaGal also by interactions with the 6th α-helix), but also the 7th β/α-loop through π–π interactions. This adjacent loop contains the disulphide bridge for the fungal galactanases and the calcium ion in BlGal and IaGal; it is much shorter in BtGal. The aromatic cluster in IaGal also interacts with an aromatic residue in the 8th β/α-loop, previously indicated as a stability hotspot in the family, which in case of IaGal and BlGal contains the − 3 subsite. The π–π interactions at the 8th β/α-loop could therefore be particularly important for general stabilization of GH53 enzymes. Because the main aromatic cluster at the 6th β/α-loop is highly structurally conserved in the family, we propose that the aromatic clusters of IaGal can inspire stabilizing strategies generally applicable in GH53 in further studies.

### Product profiles of native GH53 galactanases

This study obtained detailed degradation patterns by combining results from HPAEC-PAD and the reducing end PAHBAH assay (Figs. 2 and 8). As expected, the fungal galactanases were able to degrade G3. The accumulation of G1 and G2 is also expected and supported by an X-ray crystal structure of the fungal galactanase AaGal binding galactobiose unproductively at the − 1 and − 2 binding subsites [23]. The pattern of IaGal therefore proved highly distinct from the fungal galactanases as it was not able to degrade G3. This difference in degradation profile can be ascribed to the additional − 3 binding subsite contained in IaGal.

BlGal was likewise unable to degrade G3 when incubated with G4 [20] and lupin galactan in this study. This inability was ascribed to non-productive binding to the − 2 to − 4 binding subsites based on degradation results and X-ray crystal structures with a G3 unit bound at these binding subsites [20]. We believe that IaGal binds G3 non-productively from the − 1 to − 3 subsites.

As IaGal and BlGal both degrade to G3, the differences in their degradation patterns at high DoH are therefore diminished. The only difference at high DoH is the increased amount of G2 produced by IaGal. This could signify a slightly higher tendency for IaGal to bind G4 between the + 2 and − 2 binding subsite or it could also be due to differences in the initial degradation. IaGal does produce relatively large amounts of G > 5 fragments initially which is still present in significant quantities at a DoH of 11%. This is not observed for BlGal though it is noted that the first chromatogram is obtained with 12% DoH. The lack of observed G > 5 fragments for BlGal is likely due to a higher affinity for larger fragments by virtue of having more substrate subsites, and the preferential binding and degradation of such fragments would prevent them from accumulating. Although Lys282 in all published crystal structures blocks binding at the theoretical − 5 binding subsite, our MD simulations show Lys282 adopting a non-blocking position after approximately 60 ns (Additional file 1: Figure S4). Lys282 is a part of the 7th β/α-loop, which is stabilized by the coordinated calcium ion and does not appear to be affected by crystal contacts.

### Engineering of product profile in IaGal

Rationally designed IaGal variants were produced with altered binding sites for the non-reducing end of the substrate. The *T*_m_ of the variants showed only a slight decrease compared to the WT. For the subsite-deleted variant 4 this could directly be caused by the removal of the π–π interactions between His305 and the deleted Trp364. From structural analysis, it is deduced that the loop inserted in variant 1 and 2 contains relatively few stabilizing effects of the nature that is analysed in this study in the native BlGal structure. It contains one π–π interaction between Tyr355 and Tyr81, one salt bridge between Asp356 and Lys120 and one cation-π interaction between Tyr355 and Lys122. In the IaGal structure, the residues corresponding to Tyr81 and Lys120 have the same orientation as in BlGal and should therefore be able to make the same interactions in variant 1 and 2. The residue corresponding to Lys122 in IaGal is, however, a serine and the cation–π interaction would thus not occur.

The aim of the binding site engineering in IaGal was to obtain variants with altered degradation profiles, which was successful, but as often the case this comes at the price of slightly lower catalytic efficiency. The degradation profiles of the subsite-deleted variants showed that the mutants successfully mimic the fungal galactanases as they were able to degrade G3 indicating the intended weakening of the − 3 binding subsite. Additionally, both subsite-deleted variants were able to perform trans-glycosylation as the fungal ‘parents’. The variants, however, differed by accumulating lower amounts of G5 than MtGal and HiGal.

The degradation patterns of the subsite-extended variants did not quite resemble the degradation pattern of BlGal nor IaGal. Both variants accumulate more G > 5 fragments and less G4 fragments than the wild-type galactanases at low DoH. At high DoH, a larger amount of G1 is accumulated for the variants compared to IaGal and BlGal.

The failure to mimic faithfully the BlGal degradation pattern in the subsite-extended mutants could be due to difficulties at reconstructing interfaces in loop grafting, or specifically with adding a loop from a mesophilic structure on a hyperthermophilic structure, causing hyper-flexibility of the loop and the − 4 subsite not to be appropriately formed. The homology modelling and the following simulations do, however, indicate that the added tryptophan residue constituting the − 4 binding subsite does assume the intended conformation in both variant 1 and variant 2. Another explanation is that the − 4 subsite is successfully introduced, but that addition of the − 4 subsite is not sufficient to produce the G4 accumulation seen at low DoH for BlGal. As described before, Lys282 in the BlGal structure appears to be blocking the hypothetical − 5 binding subsite, which could contribute to this pattern. In contrast, no residues are blocking in the IaGal structure, nor should any residues introduced by the addition of the loop be blocking. If the exact degradation pattern of BlGal is needed but a higher thermostability of the enzyme is desired, the structural analysis of IaGal presented here suggests the alternative strategy of introducing more stabilizing π–π interactions in BlGal. We propose that an A280F mutation to mimic F283 of IaGal which is labelled 3 in Fig. 9B or a V369W mutation to mimic W346 in IaGal (labelled residue 4 in the same figure) could be a strategy for stabilization.

Using rational design in bioengineering to add or alter enzyme functionalities is challenging. This has mainly been successful for single-point mutations or double mutations as in Huang et al*.* [40] and Hekmat et al*.* [41] where β-mannanases were altered in terms of degradation pattern or in terms of catalytic efficiency. However, despite the decrease in catalytic efficiency of the IaGal variants, our results show that engineering of the substrate binding site of IaGal retained the hyperthermostability. These new variants could act during high-temperature pre-treatment in food and feed applications and an added advantage is the possibility of tailoring the galactan depolymerization and final population of oligomers, a crucial parameter for microbial fermentation in an ever-growing industrial segment.

## Conclusion

The first structure of a hyperthermophilic GH53 galactanase is presented in this article.

It has a temperature activity optimum of at least 95 °C, which is largely ascribed to an increased amount of salt bridges, helix stabilization, cation-π and π–π interactions compared to other GH53 galactanases with known structures. A cluster of partly conserved π–π interactions centred around the 6th β/α-loop offers great opportunities to inspire stabilizing strategies for other members of the GH53 family and some other Clan GH-A enzymes.

The substrate binding groove has intermediate structural features between the fungal endo-β-1,4-galactanases and the previously characterized BlGal, in terms of binding subsites for the non-reducing end of the substrate, which is reflected in the product profile of the enzyme. The degradation pattern of the IaGal appears to be reminiscent of that of BlGal but with small differences, particularly in the capability of IaGal to produce larger fragments; BlGal produces little to no product larger than galactotetraose, which accumulates at low DoH. Variants were designed to resemble BlGal or fungal galactanases in terms of degradation profiles. All retained high thermostability, though slightly lower than the parent. The subsite-deleted variants were highly successful in mimicking the fungal patterns as they obtained the ability to both degrade galactotriose and produce trans-glycosylation products. The subsite-extended variants were unique in terms of degradation patterns as they neither resemble BlGal nor IaGal. Homology models and subsequent molecular dynamic simulations does, however, indicate correctly grafted loops in both subsite-extended variants. These results indicate that engineering of the substrate binding site of a hyperthermostable endo-β-1,4-galactanases, and likely also other enzymes, could be a useful approach when aiming for a certain product profile.

## Methods

### Expression, purification and biochemical characterization

Bioinformatic analysis of the IaGal (UNIPROT:E0SSW8) identified an N-terminal secretion signal peptide, a catalytic domain and two C-terminal PFAM domains (PF07532 and PF09985) [42]. As described previously [43], IaGal was expressed as an extracellular C-terminal truncated enzyme of ~ 45 kDa in *Aspergillus oryzae*; i.e. the secretion signal peptide and catalytic domain of IaGal corresponding to amino acids 1 to 407.

The enzyme was purified from filtrated broth that was adjusted to a pH of 7.0 after expression. Ammonium sulphate was added to a final concentration of 1.0 M and loaded onto a 70 mL of Phenyl Sepharose™ 6 Fast Flow column (XK50, Pharmacia) equilibrated with 1.0 M ammonium sulfate, 12.5 mM HEPES pH 7.0. The bound proteins were batch eluted with 12.5 mM HEPES pH 7.0 followed by 50% EtOH in elution buffer. Fractions were collected and analysed by SDS-PAGE. The fractions were pooled and applied to a 725-mL Sephadex™ G-25 column (XK50) equilibrated in 25 mM HEPES pH 7.0 followed by a Kronlab (12 × 125 mm) column with 20 mL SOURCE™ 15Q column equilibrated in 12.5 mM HEPES pH 7.0. Bound proteins were eluted with a linear gradient from 0–500 mM NaCl over 20 CV.

The four variants were made by site-directed mutagenesis and expressed as IaGal. The four variants were purified as the native except for a few differences. The variant broths were pH adjusted to 5.8 and heated to 70 °C for 30 min followed by a 0.22-µm cup filtration. The linear gradient in the SOURCE™ 15Q column was 0–1000 mM NaCl and the variants were additionally purified on a Superdex 75 column (XK 26/60) by eluting with a constant flow of HEPES pH 7.0 and 150 mM NaCl.

The pH and temperature activity profile of IaGal, HiGal and BlGal were measured in triplicates using the PAHBAH colorimetric reducing end assay (described in detail below) [44] with lupin galactan (Megazymes, P-GALLU) as substrate. The pH activity profiles were measured by incubating between 2 ⋅ 10^−3^ mg ml^−1^ and 33 ⋅ 10^−3^ mg ml^−1^ IaGal with substrate for 15 min at 40 °C in a buffer of 100 mM glycine, 100 mM acetic acid, 100 mM HEPES, 50 mM KCl and 2 mM CaCl_2_. The pH was varied by adding HCl or NaOH.

The temperature activity profiles were measured as the pH activity profile and in the same buffer, but at a stable pH of 5.0 and varying temperatures between 40 °C to 95 °C for IaGal and 30 °C to 95 °C for HiGal and BlGal.

The thermal denaturing midpoints (*T*_m_) of IaGal and the variants were measured using a MicroCal VP-Capillary differential scanning calorimetry (DSC) instrument (Malvern Panalytical). The proteins were diluted to 0.5 mg ml^−1^ in a buffer of 50 mM NaOAc pH 5, and the differential scanning calorimetry was measured between 20–120 °C using a scan rate of 200 °C h^−1^.

The PAHBAH colorimetric reducing end assay was conducted by adding 15 µl from each reaction samples between enzyme and substrate to solutions of 4.8 mg ml^−1^ 4-hydroxybenzhydrazide, 56 mM K—Na-tartrate and 0.16 M NaOH in 96-well PCR plates (Thermo Fisher Scientific, AB0700). These solutions were incubated at 90 °C for 10 min followed by a cooling process and a hold at 20 °C for at least 2 min. The 405 nm absorbance was measured on a SpectraMax 190 spectrophotometer (Molecular Devices) and the reducing ends were quantified using external standard curves of galactose.

Degradation patterns of IaGal, the variants and the reference enzymes BlGal, MtGal and HiGal were obtained from a seven-step threefold enzyme dilution series. The highest concentration of IaGal’s and BlGal’s dilution series was 0.056 mg ml^−1^ while the highest concentration of the variants was 0.100 mg ml^−1^. The highest concentrations of MtGal’s and HiGal’s dilution series were 0.010 mg ml^−1^ and 0.020 mg ml^−1^, respectively. The enzymes were incubated in 96-well microtiter plates (Thermo Fisher Scientific) with 2.0 mg ml^−1^ lupin galactan (Megazymes, CAS: 9037-55-2) in a buffer of 50 mM NaOAc pH 5.0 and 0.01% Triton X-100 for 30 min. IaGal and the variants were incubated at 70 °C while the fungal galactanases and BlGal were incubated at 40 °C. Following the incubation 15 µl were extracted for a PAHBAH colorimetric assay while the rest was filtered through Vivaspin 500 (5000 MWCO PES, Sartorius) spin filters to exclude the enzymes from the final sample. The samples were analysed using a HPAEC-PAD ICS-3000 instrument (Dionex/Thermo Fisher), with a CarboPac PA1 column (4 × 250 mm, P/N 35391, Dionex) and a PA1 guard column (4 × 50 mm, P/N 43096, Dionex). A gold electrode with a reference Ag/AgCl electrode was used for detection.

Two eluents were used to elute the analytes. Eluent A consisted of 100 mM NaOH (Dionex graded, Honeywell, cat 415413) while eluent B consisted of 100 mM NaOH and 500 mM NaOAc. The gradient was as following: 0–10 min 0–9.0% eluent B (100–91.0% eluent A), 10–15 min 9.0% eluent B (91.0% eluent A), 15–25 min 9.0–14.7% eluent B (91.0–85.3% eluent A). This elution procedure was followed by flushing and re-equilibration of the columns.

The peaks were manually integrated in Chromeleon v7.0 software (Thermo Fisher Scientific), and the amounts of galactose, galactobiose, galactotriose, galactotetraose and galactopentaose (henceforth referred to as G1, G2, G3, G4 and G5, respectively) were quantified using external standard curves.

For the determination of Michaelis–Menten parameters, preheated substrate solutions with concentrations ranging from 0 to 1.75 mg ml^−1^ of lupin galactan (Megazymes, CAS: 9037-55-2) in a buffer of 50 mM NaOAc pH 5.0 and 0.01% Triton X-100 were incubated with the enzymes. The final concentration of IaGal was 1.0$\cdot 1{0}^{-3}$ mg ml^−1^, the variants were tested at 4.0$\cdot \hspace{0.17em}1{0}^{-3}$ mg ml^−1^ and BlGal and MtGal were tested at 0.50 $\cdot \hspace{0.17em}1{0}^{-3}$ mg ml^−1^. The solutions with IaGal or variants were incubated at 70 °C while the BlGal and MtGal were incubated at 30 °C. 15 µl samples were withdrawn at the start, after 150 s, after 300 s and after 450 s. The product formation was quantified using the PAHBAH colorimetric assay as described previously. The experiments were done in triplicates. Michaelis–Menten curves were fitted to the data using Solver in Excel and the 95% confidence interval was calculated as described in [45].

### Preparation of synthetic galactooligosaccharide standards

The β-1,4-linked galactooligosaccharides were prepared according to published procedures: galactotriose [46], galactotetraose [47] and galactopentaose [48].

### Crystallization, data collection, structure determination and analysis

Crystallization of IaGal was performed using the vapour diffusion method in MRC 2-drop plates (Molecular Dimensions) set up by an Oryx-8 robot (Douglas Instruments). The protein was initially screened using JCSG + (Molecular Dimensions), Morpheus (Molecular Dimensions) and Index (Hampton Research) screens. An initially poor hit from JCSG + was improved using Additive Screen (Hampton Research) and 2D optimization. The final hit was obtained at room temperature with a drop size of 0.3 µl with a protein:reservoir ratio of 1:1. The reservoir solution consisted of 4% w/v 1,6 hexanediol, 20 mM magnesium chloride, 0,1 M HEPES pH 7,5 and 22% w/v polyacrylic acid 5100 sodium salt, and the protein concentration was 9.7 mg ml^−1^ in a buffer of 10 mM HEPES pH 7.0 and 100 mM NaCl.

Data were collected at the microfocus beamline ID23-2 at the ESRF [49] from a multiple needle crystal. The data were collected with fine φ-slicing (0.05° per frame over 112° of data) using a beam size of 10 µm. Integration and scaling was done using XDS [50] via XDSAPP 2.0 [51] to a resolution of 2.65 Å and was then converted to mtz format with XDSCONV.

The structure was solved by molecular replacement using the automated pipeline MrBUMP [52]⁠. A solution was found with molrep from a model generated from the several available *Bacillus subtilis* structures. The solution had an *R*_free_ = 41% and *R*_work_ = 36% after an initial automated refmac5 run, which is a part of the MrBUMP routine. Several rounds of model re-building in *Coot* [53]⁠ and refinement with phenix.refine [54] resulted in a final structure with *R*_free_ = 25.7% and *R*_work_ = 20.8% (see Additional file 1: Table S1 for statistics describing data collection, processing and refinement).

To determine the structural features responsible for the increased thermostability of IaGal several intramolecular interactions were examined. For comparison, BlGal (PDB ID 1R8L), BtGal (PDB ID 6GP5), AaGal (PDB ID 1FOB), EnGal (PDB ID 4BF7), HiGal (PDB ID 1HJQ) and MtGal (PDB ID 1HJS) were used. The salt bridges were identified by measuring distances from positively charged nitrogen atoms of the sidechains in residues Arg, His and Lys, to the carboxylic acid oxygens of residues Glu and Asp. The cut-off was set to 4.0 Å and the interactions were quantified using an in-house script (http://github.com/TobiasTandrup/Ion-pairs). Hydrogen bonds within the protein were identified using HBOND (v. 1.1) [55], with a 3.5 Å cut-off. The π–π interactions were identified by measuring the distances from the middle of the aromatic rings of residues Phe, Trp, Tyr and His. The cut-off was set to 7.2 Å based on findings in Zhao et al*.* [56]. Each positive π–π interaction was visually checked to devaluate spurious interactions. The dipole stabilization/destabilization of the α-helices involved in the barrel formation was quantified as in [16, 57, 58]. The helices were defined using PROMOTIF [59]. The first three C-terminal and N-terminal residues of the helices were inspected for positively charged residues (Lys, Arg and His) and negatively charged residues (Glu and Asp). The α-helix dipole is stabilized by positively charged residues at the C-terminus and de-stabilized by negatively charged residues at the same position, and vice-versa for the N-terminus of the helices. A helix containing more stabilizing than de-stabilizing effects would be considered stable. The cation-π interactions were identified using the CaPTURE program [37]. ProteinVolume (v.1.3) [60] was used to calculate the volume and the packing density of the proteins using volume probes ranging from 0.080 to 0.020 Å in radius, while Pymol was used to calculate the surface area. Pearson correlation coefficients were calculated with the stats package in R 4.0.0 [61].

### Homology modelling and molecular dynamic simulations

Homology models of the variants were created using UniProt [62] for the sequence alignment and Modeller v. 9.25 [63] to create the models. For the models of variant 1 and 2 both BlGal (PDB ID 1R8L) and IaGal (PDB ID 7OSK) were used as templates. For the model of variant 4 MtGal (PDB ID 1HJS) and IaGal were used as templates while the model for variant 3 is only based on IaGal. The calcium ion was preserved in all models. The best model from each variant was chosen based on the DOPE score, GA341 score and visual inspection. In VMD v. 1.9.3 [64] the variant models, IaGal (PDB ID 7OSK, Chain A), BlGal (PDB ID 1R8L Chain A) and MtGal (PDB ID 1HJS Chain A) were placed in the centre of cubic water boxes with 15 nm to each edge of the box and the charge was balanced with Na^+^ ions. The TIP3P water model was used. The energy of the system was minimized over 1000 iterations using the default NAMD minimizer and the system was reheated to 300 K. The movement of the atoms were simulated over 90 ns in 2 fs timesteps at 300 K with NAMD v.2.12 [65] using CHARMM36 force field [66]. In VMD the frames of the simulations were aligned to their first frame before the simulations were analysed.

## Supplementary Information

**Additional file 1:** Supplementary Tables and Figures.

## Data Availability

The structure presented in this paper has been deposited in the Protein Data Bank (PDB) with the following accession code: 7OSK. Materials will be made available by the corresponding authors upon reasonable request.

## Abbreviations

AZCL: Azurine crosslinked
BlGal: Endo-β-1,4-galactanase from *Bacillus licheniformis*
BtGal: Endo-β-1,4-galactanase from *Bacteroides thetaiotaomicron*
DoH: Degree of hydrolysis
DSC: Differential scanning calorimetry
EnGal: Endo-β-1,4-galactanase from *Aspergillus nidulans*
GH53: Glycoside hydrolase from the family 53 in the CAZy database
G1: Galactose
G2: Galactobiose
G3: Galactotriose
G4: Galactotetraose
G5: Galactopentaose
G > 5: Galactooligosaccharides larger than galactopentaose
HiGal: Endo-β-1,4-galactanase from *Humicola insolens*
HPAEC-PAD: High-performance anion exchange chromatography coupled with pulsed amperometric detection
IaGal: Endo-β-1,4-galactanase from *Ignisphaera aggregans*
MtGal: Endo-β-1,4-galactanase from *Thermothelomyces thermophile*
PAHBAH: 4-Hydroxybenzhydrazide
RMSF: Root mean square fluctuation
WT: Wild type
