# Supplementary material for: Engineering the substrate binding site of the hyperthermostable archaeal endo-β-1,4-galactanase from Ignisphaera aggregans
Source: Biotechnol Biofuels. 2021 Sep 16;14:183. doi: 10.1186/s13068-021-02025-6 (PMC8447715; doi:10.1186/s13068-021-02025-6)
Supplement: Supplementary file 1 — Additional file 1: Supplementary Tables and Figures. [file 13068_2021_2025_MOESM1_ESM.pdf]

Additional file 1: Supplementary Tables and Figures

for ’Engineering the substrate binding site of the hyperthermostable archaeal endo β-1,4-galactanase from *Ignisphaera aggregans’*

Sebastian J. Muderspach, Folmer Fredslund, Verena Volf, Jens-Christian Navarro Poulsen, Thomas H. Blicher, Mads Hartvig Clausen, Kim Krighaar Rasmussen, Kristian B. R. M. Krogh, Kenneth Jensen & Leila Lo Leggio

Table S1. Data collection, processing and refinement. Values in outer resolution shells are given in parentheses. *Ramachandran plot was generated using PROCHECK [64] in the CCP4 suite.

PDB ID	7OSK
Diffraction source	ESRF; ID23-2
Wavelength (Å)	0.873
Temperature (K)	100
Detector	PILATUS 6M
Crystal-detector distance (mm)	248.46
Rotation range per image (°)	0.05
Total rotation range (°)	100
Exposure time per image (s)	0.05s
Space group	$P2_12_12$
a, b, c (Å)	118.0, 65.3, 94.8
α, β, γ (°)	90, 90, 90
Mosaicity (°)	0.479
Resolution range (Å)	44–2.65 (2.82–2.65)
No. of reflections	78294 (10136)
No. of unique reflections	21698 (3329)
Completeness (%)	98.5 (95.3)
Redundancy	7.72 (6.52)
⟨I/σ(I)⟩	7.62 (1.96)
$CC_{1/2}$	98.0 (69.8)
R_{meas} (%)	19.1 (77.2)
Overall B factor from Wilson plot (Å^2)	26.75
**Refinement**	
Final R_{cryst} (%)	20.8
Final R_{free} (%)	25.7
No. of non-H atoms	5925
Protein	5788
Ligand	60
Solvent	77
R.m.s. deviations	
Bonds (Å)	0.003
Angles (°)	0.73
Average *B* factors (Å^2)	23.50
Protein	23.54
Ligand	23.95
Solvent	19.64
Ramachandran plot*	
Favoured regions (%)	93.58
Additionally allowed (%)	6.28
Outliers (%)	0.14

Table S2. Analysis of structural characteristics in the GH53 potentially related to thermal adaptation. PDB structures analysed were: 7OSK (IaGal), 1HJQ (HiGal), 1HJS (MtGal), 1FOB (AaGal), 4BF7 EnGal, 1UR4 (BlGal) and 6GP5 (BtGal). Helices were classified as stabilized/destabilized based on charged residues interactions with the helix dipoles, as in [16]. The Pearson correlation of the various features with the activity temperature optimum is indicated. BtGal, for which the temperature optimum has not been experimentally measured, is included in the calculations outside the brackets and excluded inside the brackets.

	**IaGal**	**HiGal**	**MtGal**	**AaGal**	**EnGal**	**BlGal**	**BtGal**	**Pearson correlation**
Temperature activity optimum [°C]	95	70	65*	50*	49†	40	37‡	
T_m [°C]	104.5	75	N/A	60.9	N/A	53.4	N/A	
Salt bridges per residue	0.067	0.045	0.048	0.036	0.045	0.056	0.086	-0.05 (0.61)
Cation- π interactions per residue	0.022	0.024	0.024	0.018	0.018	0.013	0.016	0.74 (0.70)
π - π interactions per residue	0.114	0.087	0.096	0.090	0.078	0.076	0.064	0.94 (0.94)
Number of stabilized α-helices	6	5	6	2	4	4	7	0.26 (0.70)
Number of destabilized α-helices	1	0	0	2	0	4	0	-0.25 (-0.45)
Difference in number stabilized to destabilized α-helices	5	5	6	0	4	0	7	0.29 (0.67)
Proline to Glycine ratio	0.63	0.77	0.77	0.54	0.46	0.35	0.63	0.49 (0.67)
Hydrogen bonds per residue	1.03	1.10	1.11	1.12	1.16	1.05	1.07	-0.33 (-0.48)
Surface to Volume ratio [$Å^{-1}$]	0.82	0.76	0.76	0.76	0.82	0.75	0.76	0.53 (0.50)

*[16]

†[24]

‡This value is not measured experimentally but hypothesized given that the source bacteria live in the human gut.

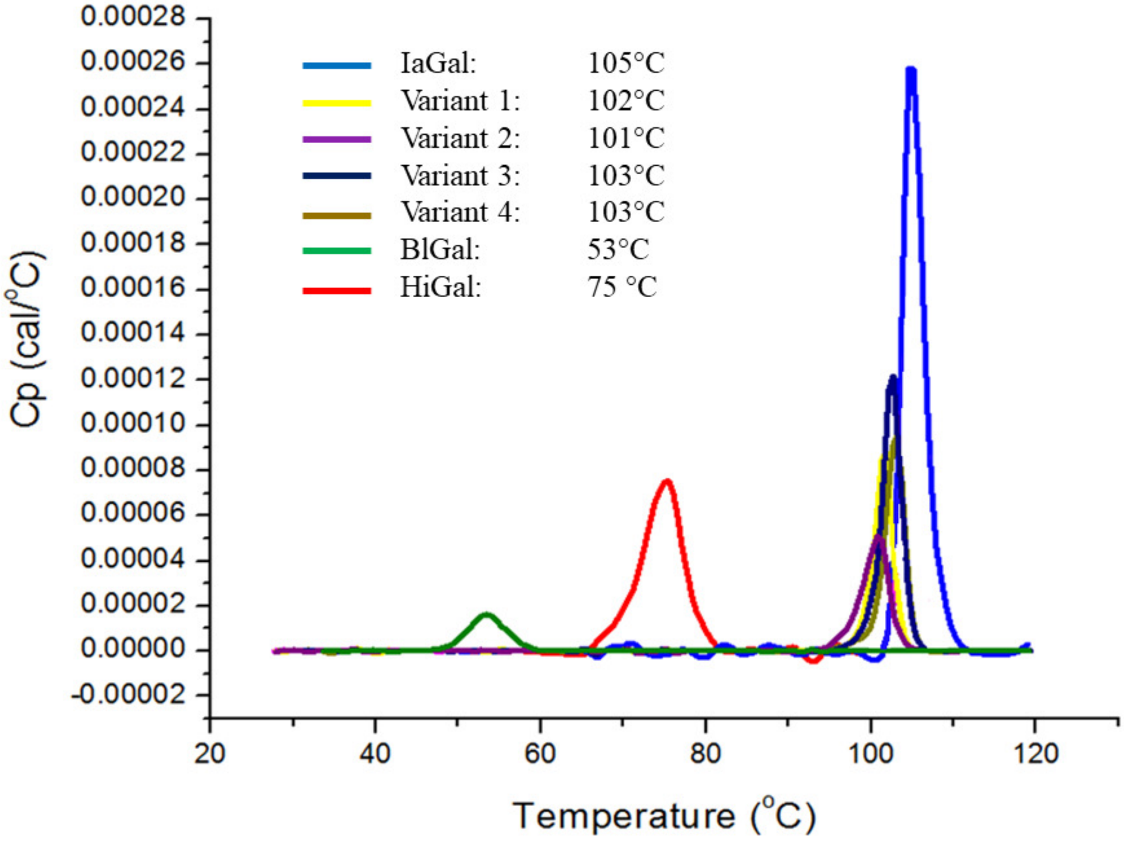

Figure S1. Differential scanning calorimetry graphs for BlGal, HiGal, IaGal and variants of IaGal. The graphs were measured using protein concentrations of 0.5 mg/ml in a buffer of 50 mM sodium acetate pH 5.0.

```
IaGal     1    ----------PVIINPVPG-------LPVDFIRGVDASEAPWIIELGGKYYDENGVERDL    43
MtGal     1    ----------------------------ALTYRGVDWSSVVVEERAGVSYKNTNGNAQPL    32
HiGal     1    ----------------------------ALQYKGVDWSSVMVEERAGVRYKNVNGQEKPL    32
AaGal     1    ----------------------------ALTYRGADISSLLLLEDEGYSYKNLNGQTQAL    32
EnGal     1    -----------MILSSLLPLSLVTLTSAALTYRGADISSLLIEEDSGVAYKNLNGETQAF    49
BlGal     1    AHRDSGTAKSGLYVEKVSG-------LRKDFIKGVDVSSIIALEESGVAFYNESGKKQDI    53
                                               :*.* *.       *  : : .*  : :

IaGal    44    LDILKENGVNWIRLRVWNDPYDEQGRPYGGGNCDLPRMTDFAAKAKAKGFGVLIDFHYSD   103
MtGal    33    ENILAANGVNTVRQRVWVNPAD--------GNYNLDYNIAIAKRAKAAGLGVYIDFHYSD    84
HiGal    33    EYILAENGVNMVRQRVWVNPWD--------GNYNLDYNIQLARRAKAAGLGLYINFHYSD    84
AaGal    33    ETILADAGINSIRQRVWVNPSD--------GSYDLDYNLELAKRVKAAGMSLYLDLHLSD    84
EnGal    50    ELILANNGVNSIRQRIWVNPSD--------GSYNLEYNLELAKRVQDAGMSVYLDLHLSD   101
BlGal    54    FKTLKEAGVNYVRVRIWNDPYDANGNGYGGGNNDLEKAIQIGKRATANGMKLLADFHYSD   113
                  *   *:* :* *:* :* *        *. :*     :. :.   *: :  ::* **

               Subsite -2                                    Catalytic acid/base
IaGal   104    WWADPSKQSKPKAWANLSYPELVEAVYNWTYNALKYMAEHNALPDMVQIGNEINNGFLWP   163
MtGal    85    TWADPAHQTMPAGWP-SDIDNLSWKLYNYTLDAANKLQNAGIQPTIVSIGNEIRAGLLWP   143
HiGal    85    TWADPAHQTTPAGWP-SDINNLAWKLYNYTLDSMNRFADAGIQVDIVSIGNEITQGLLWP   143
AaGal    85    TWADPSDQTTPSGWSTTDLGTLKWQLYNYTLEVCNTFAENDIDIEIISIGNEIRAGLLWP   144
EnGal   102    TWADPGDQATPSGWSTTDIDTLAWQVYNYTLDVCNTFAENNVAVEIVSIGNEIRNGLLHP   161
BlGal   114    FWADPAKQKAPKAWANLNFEDKKTALYQYTKQSLKAMKAAGIDIGMVQVGNETNGGLA--   171
                ****..*  * .*   .       :*::* :  : :   .    ::.:***   *:

IaGal   164    DGSAANWTQFVGLLKAAISAVKDVN--PNIKIVIHLAGVKAD-FYINFIDRL----INSG   216
MtGal   144    TGRTENWANIARLLHSAAWGIKDSSLSPKPKIMIHLDNGWDWGTQNWWYTNVLKQGTLEL   203
HiGal   144    LGKTNNWYNIARLLHSAAWGVKDSRLNPKPKIMVHLDNGWNWDTQNWWYTNVLSQGPFEM   203
AaGal   145    LGETSSYSNIGALLHSGAWGVKDSNLATTPKIMIHLDDGWSWDQQNYFYETVLATGELLS   204
EnGal   162    LGSTDHYDNIARLLHSGAWGVKDSSLSTTPKILFHLDNGWDWDAQKYFYDTVLATGTLLS   221
BlGal   172    --GETDWAKMSQLFNAGSQAVRETD--SNILVALHFTNPETSGRYAWIAETL----HRHH   223
                     : ::  *:::.  .:::     .  : .*: .             :

                                                   Catalytic nucleophile
IaGal   217    VSFDVIAISFYPYWH--GTMDDFRNLVRTLVQRYDKKILVAETAYAWTLDDSDGHPNIFG   274
MtGal   204    SDFDMMGVSFYPFYSSSATLSALKSSLDNMAKTWNKEIAVVETNWPISCPNPR---YSFP   260
HiGal   204    SDFDMMGVSFYPFYSASATLDSLRRSLNNMVSRWGKEVAVVETNWPTSCPYPR---YQFP   260
AaGal   205    TDFDYFGVSYYPFYSASATLASLKTSLANLQSTYDKPVVVVETNWPVSCPNPA---YAFP   261
EnGal   222    TDFDLIGVSYYPFYNADATLSSLKTSLTNLKSNYGKNVLVVETDWPVQCSSPE---YAFP   278
BlGal   224    VDYDVFASSYYPFWH--GTLKNLTSVLTSVADTYGKKVMVAETSYTYTAEDGDGHGNTAP   281
                .:* :. *:**::   .*:  :   : .: . :.* : *.** :

IaGal   275    SRDLEVKGGYKASIQGQASFIRDLIAALYEEGKDKALGIFYWGATWIPYPG---------   325
MtGal   261    SDV----KNIPFSPEGQTTFITNVANIVSSV--SRGVGLFYWEPAWIHNAN---------   305
HiGal   261    ADV----RNVPFSAAGQTQYIQSVANVVSSV--SKGVGLFYWEPAWIHNAN---------   305
AaGal   262    SDL----SSIPFSVAGQQEFLEKLAAVVEAT--TDGLGVYYWEPAWIGNAG---------   306
EnGal   279    SDL----SSIPFSADGQETFLGRLADTLE-D--VGGVGIYYWEPGWVDNAG---------   322
BlGal   282    KNGQTLN--NPVTVQGQANAVRDVIQAVSDVG-EAGIGVFYWEPAWIPVGPAHRLEKNKA   338
                           :  **   :  :   :       .:*::**   *:

                    Subsite -3     Subsite -4
IaGal   326    ------AGWKTG--------------EGNPWENQALFDFNGRA-LPSLKVFRLVYE------   360
MtGal   306    --------------------------LGSSCADNTMFSQSGQA-LSSLSVFQRI--------   332
HiGal   306    --------------------------LGSSCADNTMFTPSGQA-LSSLSVFHRI--------   332
AaGal   307    --------------------------LGSSCADNLMVDYTTDEVYESIETLGEL--------   334
EnGal   323    --------------------------LGSSCEDNLMVDWRDRTVRESISVFGDLAA------   352
BlGal   339    LWETYGSGWATSYAAEYDPEDAGKWFGGSAVDNQALFDFKGRP-LPSLHVFQYVDTGTPFKN   399
                                          *.   :: :.         *: .:  :
```

Figure S2. Sequence alignment of IaGal, MtGal (PDB ID 1HJS), HiGal (PDB ID 1HJQ), AaGal (PDB ID 1FOB), EnGal (PDB ID 4BF7) and BlGal (PDB ID 1UR0) calculated with Clustal Omega in Uniprot [65]. The tryptophan residues that create the platform for the -2, –3 and –4 subsites and the catalytic residues have been labelled. (*) Indicates an identical residue between all structures. (:) indicates a conserved residue while (.) indicates a semi-conserved residue.

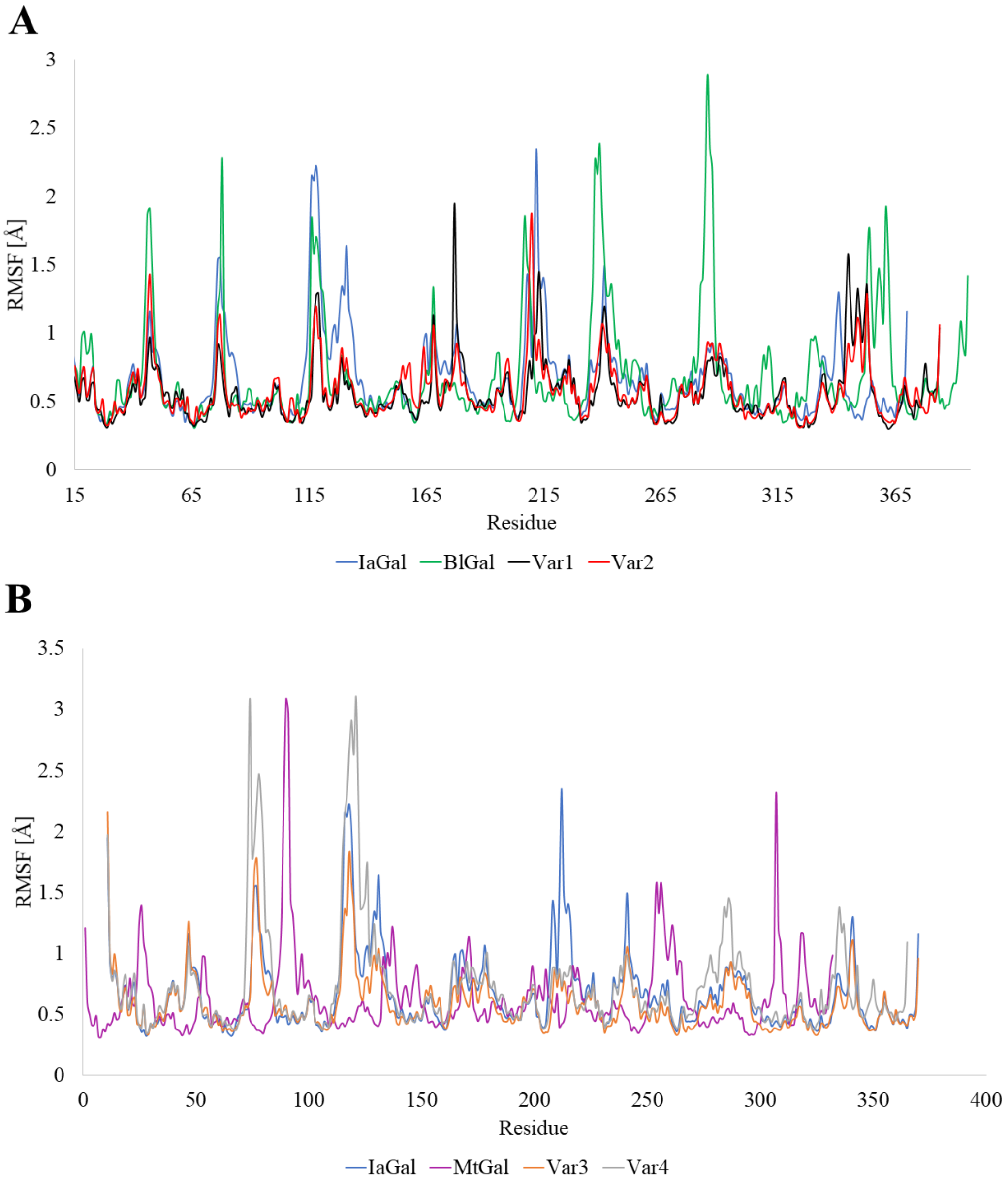

Figure S3. (A) RMSF is shown for each residue in IaGal (blue), BlGal (green), variant 1 (black) and variant 2 (red). (B) RMSF shown for each residue in IaGal (blue), MtGal (magenta), variant 3 (orange), variant 4 (grey).

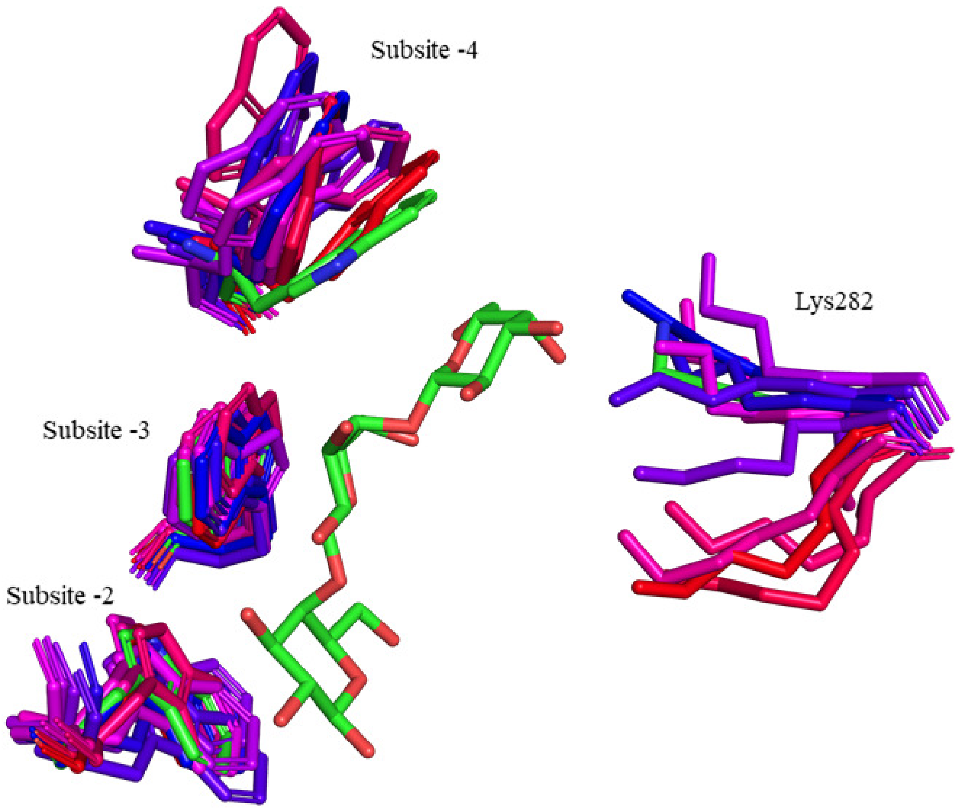

Figure S4. The overview of the potential blocking of larger substrates in BlGal. The -2, -3 and -4 binding subsite in addition to Lys282 and the G3 substrate of BlGal (PDB ID 1UR0) is shown in green. The same residues of the molecular dynamic simulation of BlGal (PDB ID 1R8L) is shown in a spectrum from blue (0 ns) to red (90 ns).
